# Integration of multiple prognostic predictors in a porcine spinal cord injury model: A further step closer to reality

**DOI:** 10.3389/fneur.2023.1136267

**Published:** 2023-03-08

**Authors:** Chao-Kai Hu, Ming-Hong Chen, Yao-Horng Wang, Jui-Sheng Sun, Chung-Yu Wu

**Affiliations:** ^1^Department of Neurosurgery, Mackay Memorial Hospital, Taipei, Taiwan; ^2^Department of Biological Science and Technology, College of Biological Science and Technology, National Yang Ming Chiao Tung University, Hsinchu, Taiwan; ^3^Graduate Institute of Nanomedical and Medical Engineering, Taipei Medical University, Taipei, Taiwan; ^4^Department of Neurosurgery, Wang Fang Hospital, Taipei Medical University, Taipei, Taiwan; ^5^Department of Pet Healthcare, Yuanpei University of Medical Technology, Hsinchu, Taiwan; ^6^Trauma and Emergency Center, China Medical University Hospital, Taichung City, Taiwan; ^7^College of Medicine, China Medical University, Yingcai Campus, Taichung City, Taiwan; ^8^College of Biomedical Engineering, China Medical University, Yingcai Campus, Taichung City, Taiwan; ^9^Department of Orthopedic Surgery, National Taiwan University Hospital, Taipei, Taiwan; ^10^Department of Electronics Engineering and Institute of Electronics, National Yang Ming Chiao Tung University, Hsinchu, Taiwan

**Keywords:** spinal cord injury, porcine model, balloon compression, motor behavior, intraspinal pressure, spine-to-spine evoked potentials, magnetic resonance imaging

## Abstract

**Introduction:**

Spinal cord injury (SCI) is a devastating neurological disorder with an enormous impact on individual's life and society. A reliable and reproducible animal model of SCI is crucial to have a deeper understanding of SCI. We have developed a large-animal model of spinal cord compression injury (SCI) with integration of multiple prognostic factors that would have applications in humans.

**Methods:**

Fourteen human-like sized pigs underwent compression at T8 by implantation of an inflatable balloon catheter. In addition to basic neurophysiological recording of somatosensory and motor evoked potentials, we introduced spine-to-spine evoked spinal cord potentials (SP-EPs) by direct stimulation and measured them just above and below the affected segment. A novel intraspinal pressure monitoring technique was utilized to measure the actual pressure on the cord. The gait and spinal MRI findings were assessed in each animal postoperatively to quantify the severity of injury.

**Results:**

We found a strong negative correlation between the intensity of pressure applied to the spinal cord and the functional outcome (*P* < 0.0001). SP-EPs showed high sensitivity for real time monitoring of intraoperative cord damage. On MRI, the ratio of the high-intensity area to the cross-sectional of the cord was a good predictor of recovery (*P* < 0.0001).

**Conclusion:**

Our balloon compression SCI model is reliable, predictable, and easy to implement. By integrating SP-EPs, cord pressure, and findings on MRI, we can build a real-time warning and prediction system for early detection of impending or iatrogenic SCI and improve outcomes.

## 1. Introduction

Spinal cord injury (SCI) can lead to permanent loss of neurological function. Furthermore, SCI can impose enormous emotional, psychological, and financial costs on affected patients and their families, as well as a substantial burden on medical facilities and society (1). A reliable and reproducible animal model of SCI is crucial to evaluate the severity, progress of injury, and extent of recovery to enable a deeper understanding of SCI and to further evaluate therapeutic approaches.

The contusion model of SCI, wherein a transient force displaces and damages the spinal cord using a weight-drop-based instrument, was first described by Allen (2) in 1911 and is a widely used model because it simulates the clinical outcomes of humans suffering from acute traumatic SCI (3). By changing the weight, height, or velocity of the impactor, varying degrees of spinal cord damage can be induced. However, the mechanism of trauma and clinical SCI may involve several etiologies, which relate to a constant spinal canal occlusion and pressure, such as degenerative disease of the vertebral column, malignancy, and infection. We believe that the compression model, in which the spinal cord is subjected to a constant pressure from a balloon or pressure pad over an extended time period, is a better way of simulating SCI in most situations. By varying the size of the balloon or compression time, different degrees of SCI can be created (4). It also allows for investigating the pathological effects of spinal cord compression and optimal timing of decompression (5, 6).

The severity of SCI should directly correlate with the degree of pressure on the spinal cord; however, the actual pressure exerted on the spinal cord is seldom considered in the literature. Current guidelines for treatment of traumatic brain injury indicate that the highest intracranial pressure (ICP) that brain tissues can withstand is 20–22 mmHg (7, 8). Exceeding this value will cause irreversible damage to the brain tissue. To the best of our knowledge, no pressure tolerance value for the spinal cord or its cellular constituents has been established.

Neurological outcomes are frequently assessed using cellular biology, behavior, neurophysiology, imaging, or cardiovascular, musculoskeletal, and respiratory parameters. The prediction accuracy can be improved by combining a greater variety of evaluation tools, thereby avoiding irreversible damage. However, studies that have integrated up to four assessment parameters simultaneously account for <1% of those reported (9).

In the present study, we tested our large animal spinal cord compression injury model induced using an implantable balloon catheter to compress the T8 level of the porcine spinal cord by balloon inflation. Multiple predictors were used to evaluate the prognosis in these pigs, allowing for a more direct comparison to be made with human SCI. Spinal cord injury severity was categorized according to post-operative pig's hindlimb behavior. An original intraspinal pressure (ISP) monitoring technique was utilized to measure the actual pressure on the spinal cord. In addition to the basic neurophysiological recording of somatosensory evoked potentials (SSEPs) and motor evoked potentials (MEPs), we introduced spine-to-spine evoked spinal cord potentials (SP-EPs) by direct stimulation, recording the spinal cord just above and below the diseased segment. Each pig's gait and spinal magnetic resonance imaging (MRI) findings were assessed postoperatively to quantify the degree of injury.

## 2. Materials and methods

### 2.1. Animals

Fourteen 15–36-week-old domestic pigs, weighing 39.5–92.0 kg, were used in the experiment. All the study procedures were reviewed and approved by the Institutional Animal Care and Use Committee of Pigmodel Animal Technology Co., Ltd. All applicable institutional and governmental regulations concerning the ethical use of animals were followed during the research course. The study protocol was designed to minimize the number of animals used and the distress caused to the animals. All behavioral and image analyses were performed by operators who were blinded to the biomechanical severity of the surgically-induced SCI.

### 2.2. Surgical procedure

The surgery was performed by a well-trained neurosurgeon. Betadine was applied preoperatively to sterilize the surgical site. Anesthesia was induced by intramuscular injection of azaperone 2–4 mg/kg, atropine 0.02–0.05 mg/kg, and a combination of zolazepam + tiletamine 5–7 mg/kg. Ventilation was maintained using an endotracheal tube at a rate of 10–12 breaths/min with a tidal volume of 12–15 mL/kg. Sedation was maintained intraoperatively using a continuous infusion of propofol at a rate of 50–100 mg/kg/min. The heart rate, respiratory rate, blood pressure, and oxygen saturation were monitored intraoperatively using standard techniques. Hydration was maintained using intravenous lactated Ringer's solution. The temperature was measured by a rectal temperature probe and maintained at 35.0–37.0°C.

**Figure 1:**
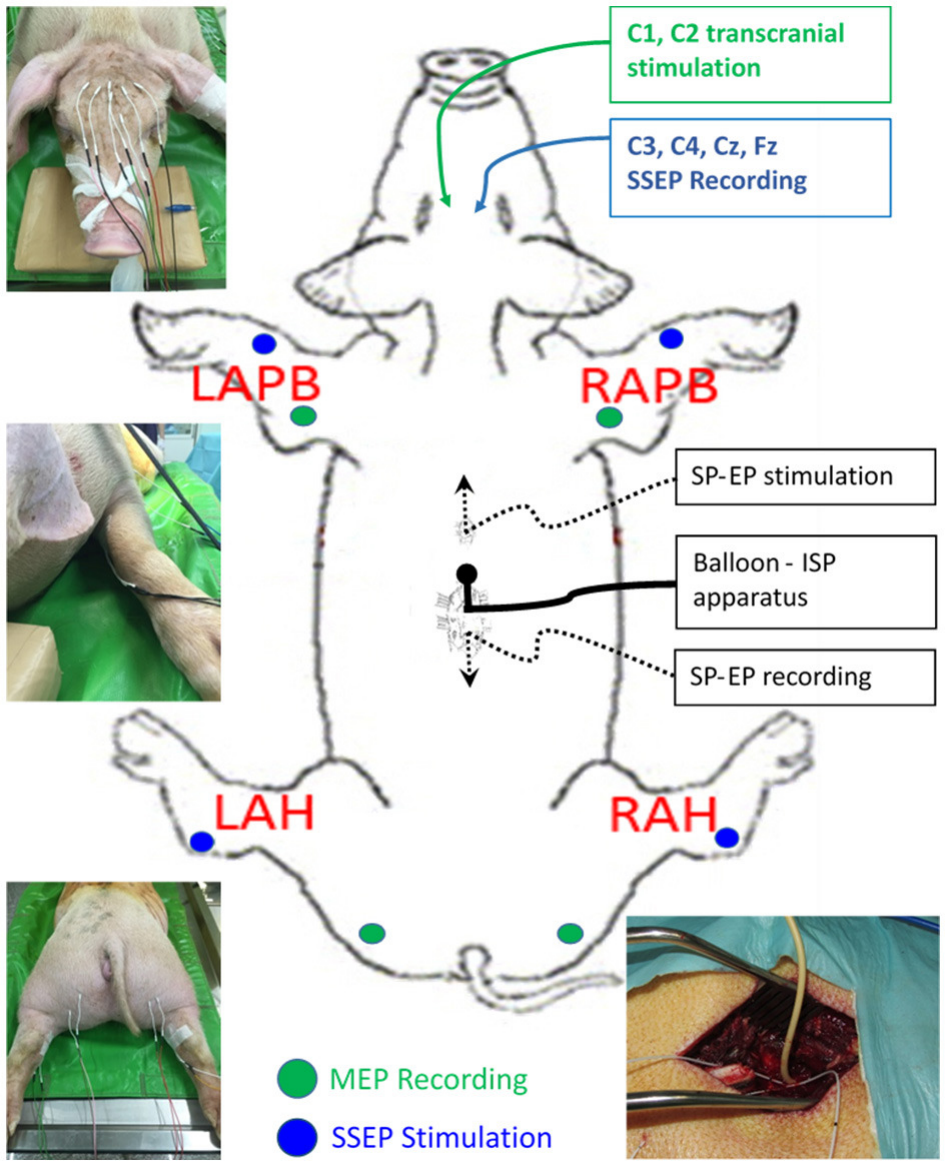
Diagram showing the experimental set-up. SSEP, somatosensory evoked potentials; MEP, motor evoked potentials.

With the pig in a prone position on the operating table, baseline SSEP and MEP data were obtained by intraoperative neuromonitoring (IONM) using a portable IONM system (Cascade Elite; Cadwell Industries Inc., Kennewick, WA, USA). Electrical stimulation and recordings were performed using subdermal needle electrodes (Neuroline 27-gauge, 12-mm Stainless Steel Ultra Sharp Needle Tip Disposable Sterile; Ambu Sdn Bhd, Penang, Malaysia). For SSEP recordings, we stimulated the median and tibial nerves on each side and recorded them at the C3 and C4 points on the cranium. Nerve stimulation was performed at 2.79 Hz with a pulse width of 200 μs and a current intensity of 25 mA for 200 sweep averages. SSEPs were recorded continuously during the entire procedure. MEPs were evoked at C1 and C2 using a train of eight monophasic constant voltage pulses at a pulse width of 50 μs, a stimulation pulse interval of 2 ms, and a stimulation power in the range of 350–442 V. Different montages and stimulation intensities were used to determine the most effective excitation for the extensor carpi radialis and biceps femoris muscles on each side (Figure 1).

**Figure 2:**
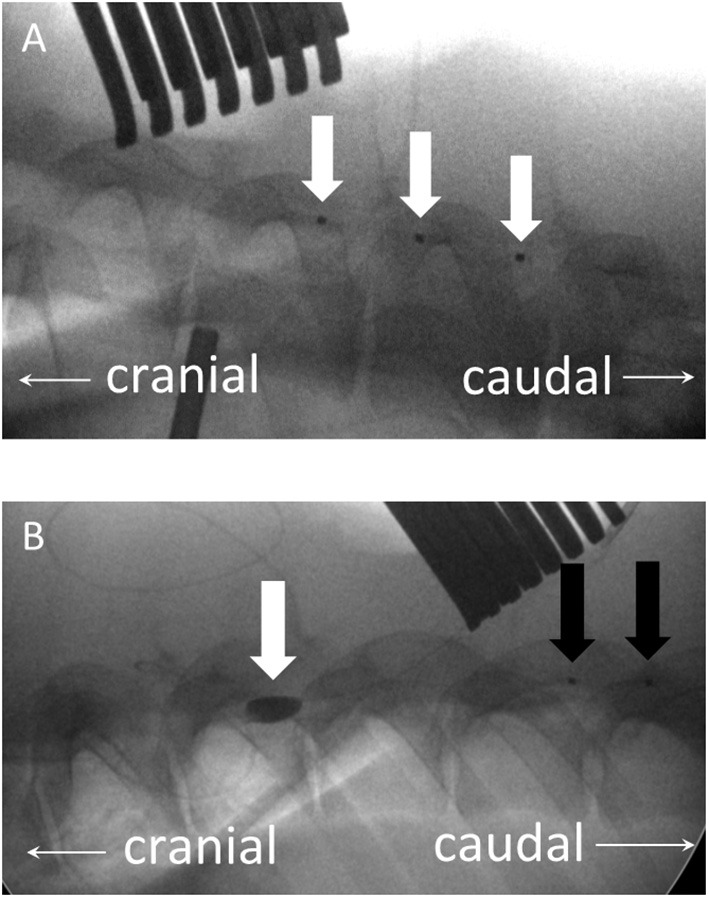
**(A)** Plain lateral radiograph showing an upstream three-contact platinum catheter electrode (D-wave electrode), inserted epidurally in the cephalic direction (white arrows) *via* a T5 flavectomy. **(B)** A downstream D-wave wire (black arrows) is inserted in the caudal direction epidurally from this laminectomy window. The balloon, which has been inflated with a contrast medium, is located at the T8 level (white arrow).

After setting up the IONM protocol, the location of T8 was confirmed with a lateral radiograph. Two dorsal midline incisions were made at approximately the T5 and T10 levels. The spinous processes, laminae, and part of the facet joints at T5 and T10 were exposed by electrocautery. Two flexible three-contact platinum catheter D-wave electrodes (CEDL-3PIDINX; Ad-Tech Medical Instruments Corporation, Racine, WI, USA) were prepared for the application of the SP-EP method. A T5 flavectomy was performed, followed by the insertion of a D-wave electrode epidurally upstream in the cephalic direction (Figure 2A, white arrows). Then, a T10 laminectomy was performed and widened to ensure that a circular window measuring at least 1.2 cm in diameter was made to expose the dura and spinal cord. A downstream D-wave wire was inserted epidurally at this laminectomy window in the caudal direction (Figure 2B, black arrows). SP-EPs were stimulated using cranial epidural electrodes at 2.79 Hz with a 200-μs pulse duration at an intensity of 1 mA for 200 sweep averages and recorded using a caudal epidural electrode.

**Figure 3:**
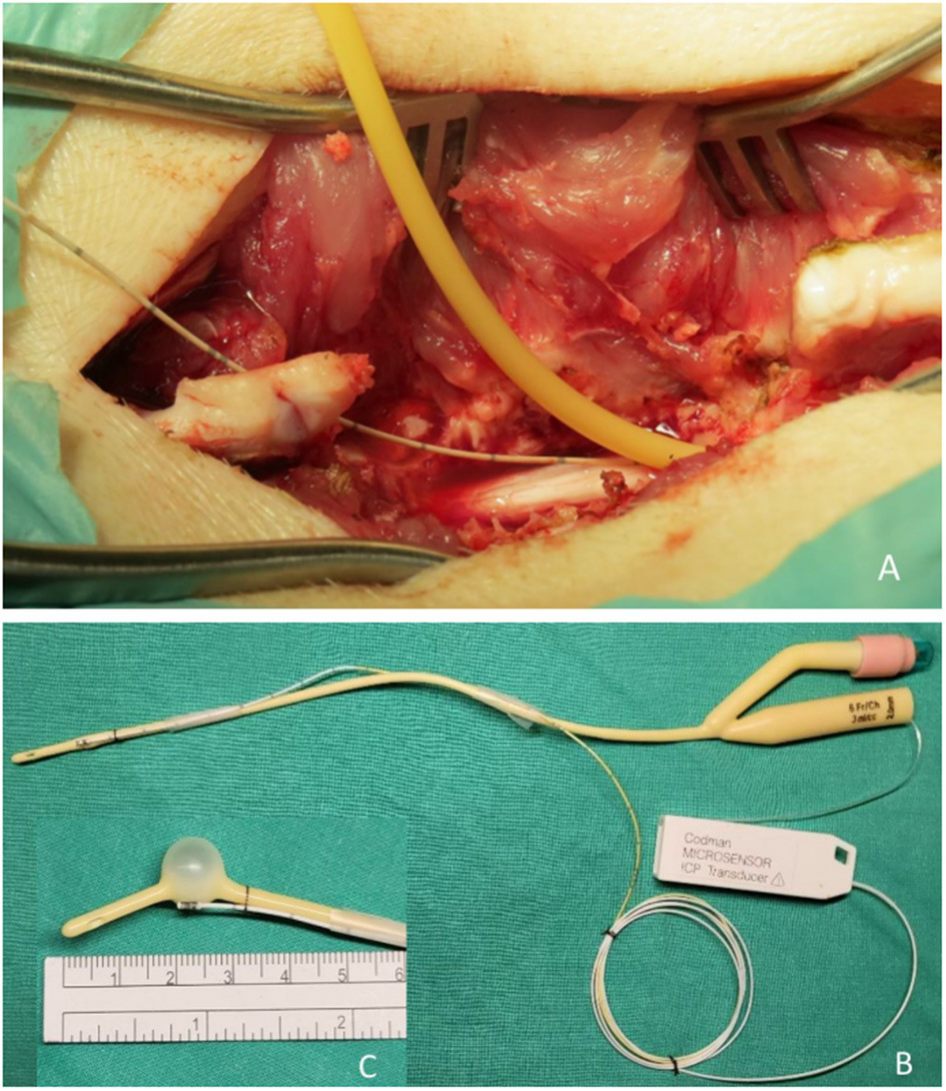
Photographs showing the surgical site. **(A)** A widened T10 laminectomy was created as the injury site. A Foley catheter with pressure sensor apparatus was implanted epidurally in the cranial direction. **(B)** A Foley balloon catheter was mounted with a pressure transducer. **(C)** The position of the pressure sensor is precisely at the center of the balloon when inflated.

The thoracic spine epidural space was then entered using a 6-Fr Foley balloon catheter. The Foley balloon had a maximal capacity of 3 mL, and the inflated balloon was 11 mm in diameter and 15 mm in length. A pressure sensor (Codman Microsensor^®^ ICP Transducer, Basic Kit; Integra LifeSciences Corp., Plainsboro, NJ, USA) was mounted to the catheter in the expansile balloon area before entering the epidural space (Figure 3). The pressure sensor had a sensor tip of 1.3 mm diameter, a bare fiber of 0.8 mm diameter, and a length of 100 cm. The measurement range was 50–250 mmHg. This device has been widely used to monitor clinical ICP in various conditions, including traumatic brain injury, intracerebral hemorrhage, and tumors. The sensor was used to measure the ISP in real-time in the area where the balloon expanded. The balloon catheter was advanced under fluoroscopic guidance until it reached the T8 level and was slowly inflated by injecting normal saline using an inflation device. The position and shape of the balloon were confirmed by fluoroscopy using a contrast medium (Figure 2B, white arrows). Each 0.2-mL increment was maintained for 5 min to obtain a stable pressure. During balloon inflation, ISP, SSEP, MEP, and SP-EP values were recorded simultaneously. Inflation was stopped at different degrees of evoked potential waveform changes, that is, complete or partial loss of SSEP, MEP, or SP-EP. The balloon catheter was removed after deflation, while the ISP sensor wire was left *in situ* to measure changes in spinal cord pressure. After wound closure, an ISP connector was fixed to the pig's skin.

### 2.3. Postoperative management

All animals received antibiotic prophylaxis with cefazolin 30 mg/kg intravenously every 8 h, which was initiated at the start of the surgery and continued for up to 24 h postoperatively. Typically, the animals were housed individually for the entire observation period. A urinary catheter was not placed routinely. The bladder was expressed manually daily to treat dysuria, and soft pads and a sling were used to prevent pressure areas.

### 2.4. Behavioral assessments

**Figure 4:**
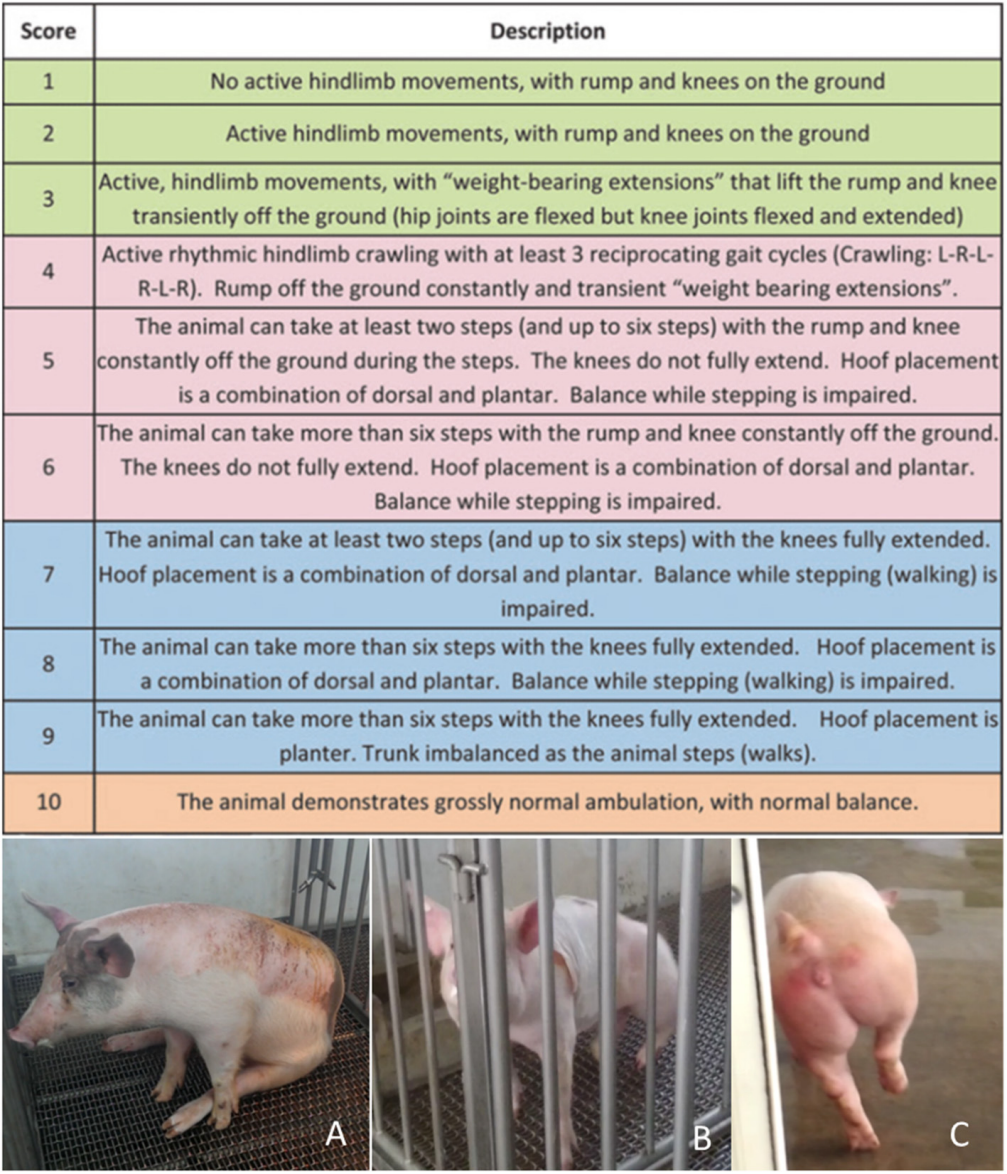
The Porcine Thoracic Injury Behavioral Scale (PTIBS). **(A)** PTIBS score 1, complete loss of active hindlimb movements. **(B)** PTIBS score 3, the rump and knee are lifted off the ground transiently with “weight-bearing extensions.” **(C)** PTIBS score 8, more than six steps can be taken with the knees fully extended but balance while walking is mildly impaired.

The gait of each pig was recorded using a video camera after spinal cord compression. Motor function was quantitatively evaluated using the Porcine Thoracic Injury Behavior Scale (PTIBS) (1). Hindlimb function values were analyzed and classified into 10 stages, ranging from no active hindlimb movements (score, 1 point) to normal ambulation (score, 10 points). PTIBS scores of 1–3 are characterized by hindlimb dragging, scores of 4–6 reflect varying degrees of stepping ability, and scores of 7–10 reflect varying degrees of walking ability (Figure 4).

The recovery status was categorized as complete, incomplete, or asymptomatic SCI (compression without postoperative symptoms) according to postoperative behavior. Complete SCI was defined as complete paralysis immediately after the operation with a PTIBS score of 1–3 points throughout the observation period. Pigs with complete SCI moved using only their forelimbs without obvious joint contracture in the hindlimbs. Incomplete SCI was defined as weakness (score, 1–7 points) immediately after the operation that improved gradually until the animal was capable of walking (score, 8–10 points). Animals that did not have hindlimb weakness after surgery despite compression and elevation of spinal pressure (PTIBS score > 8 points) were classified as asymptomatic.

### 2.5. Analysis of MRI findings

The quantification method was based on the premise that the clinical significance of a lesion in the spinal cord depends on its morphology, length, location, and area. A damaged segment of the spinal cord may exhibit three characteristics on MRI: cord swelling, as indicated by changes in the caliber of the spinal cord; cord edema, as indicated by intramedullary hyperintensity on T2-weighted (T2W) images; and cord hemorrhage, as indicated by focal isointensity to hypointensity on T2W or gradient-echo images (10).

**Figure 5:**
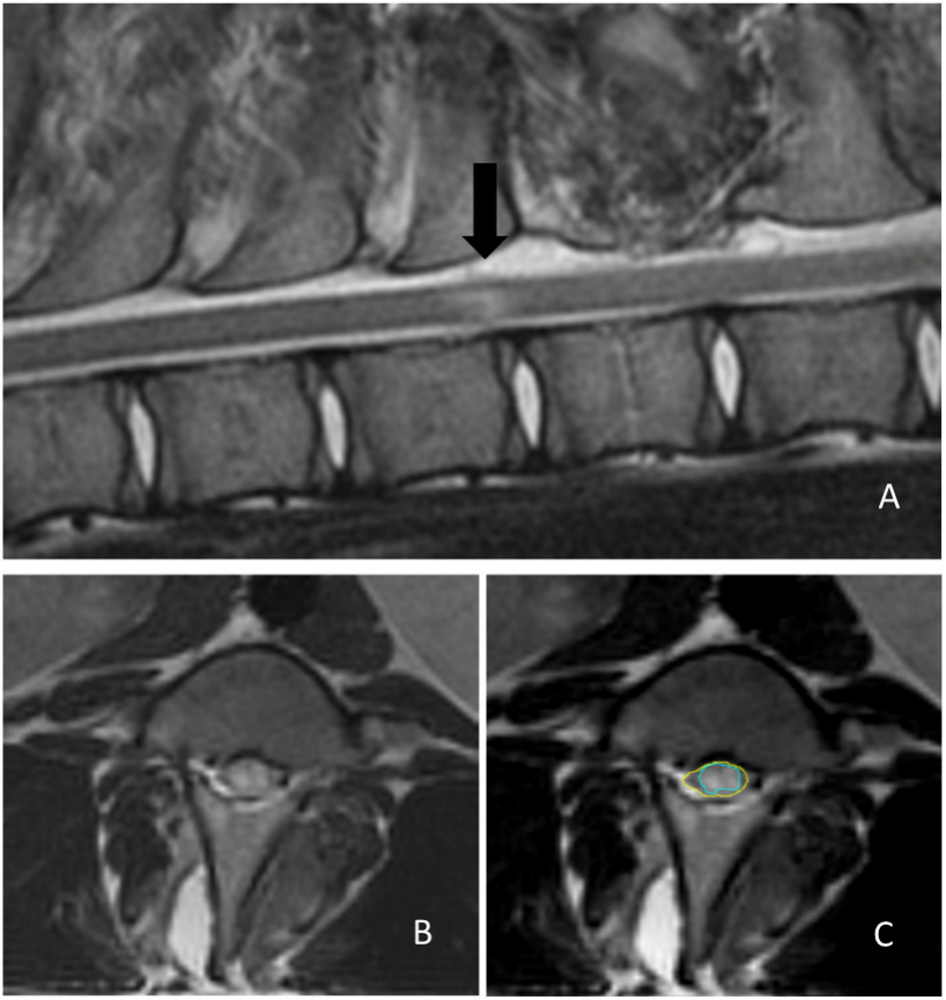
Determination of the extent of the trauma-induced region on magnetic resonance images. **(A)** Mid-sagittal T2-weighted image showing an intramedullary hyperintensity lesion (black arrow) indicating cord damage. **(B)** A segment of the maximal hyperintensity area in a transverse T2-weighted image. **(C)** The severity of the spinal cord injury is determined by comparing the hyperintense area (green circle) ratio to the total cross-sectional area of the spinal cord (yellow circle) using ImageJ software.

All pigs underwent MRI procedures on postoperative day 3 when swelling of the spinal cord tissue reached its peak. MRI examination was performed using a 1.5-T scanner (Magnetom Symphony 1.5 T; Siemens Healthcare, Erlangen, Germany) under general anesthesia with the animal in sternal recumbency. Sagittal (repetition time, 3,130 ms; echo time, 113 ms; slice thickness, 3 mm) and transverse (repetition time, 3,140 ms; echo time, 69 ms; slice thickness, 3 mm) T2W images were evaluated using a workstation with appropriate software (MicroDicom DICOM viewer, OFFIS e.V., Oldenburg, Germany). A trauma-induced region was identified by loss of integrity of the gray/white matter in the spinal cord and the appearance of an irregular higher-intensity tissue mass on T2W images. If an intramedullary hyperintensity was present on sagittal T2W images, the largest area of hyperintensity was identified (Figure 5). The presence and degree of SCI were evaluated by comparing the ratio of the hyperintensity area to the total cross-sectional area of the spinal cord at the segment of balloon compression on transverse T2W images using ImageJ software (National Institutes of Health, Bethesda, ML, USA).

### 2.6. Statistical analysis

Categorical variables are expressed as percentages and continuous variables as means and standard deviations (means ± SDs). The significance of each variable among recovery status was assessed using ANOVA test. To evaluate the relative risk associated with recovery status, univariate analysis was performed to estimate the odds ratio (OR) and 95% confidence interval (CI). All statistical analyses were performed using SAS software version 9.1 (SAS Institute Inc, Cary, NC, USA). A *P*-value < 0.05 was considered statistically significant.

## 3. Results

### 3.1. Animal characteristics and behavioral outcomes

Balloon compression was successfully performed in all 14 pigs. All pigs were female, with a mean age of 21.7 ± 7.2 (range, 14–36) weeks and a mean weight of 56.5 ± 17.1 (range, 39.5–92.0) kg (Table 1). The mean weight was 59.3 ± 22.1 kg in the complete SCI group, 49.4 ± 4.7 kg in the incomplete SCI group, and 59.3 ± 19.9 kg in the asymptomatic group. No significant between-group differences were observed in weight or age.

**Table 1:** Demographic characteristics in 14 pigs.

**Variable**	**Category**	**Total**	**Complete**	**Incomplete**	**Asymptomatic**	***P*-value**
Amount		14	4	4	6	-
Mean age (weeks)	*N*	14	4	4	6	0.692
	Mean (SD^a^)	21.7 (7.2)	21.8 (10.0)	19.1 (3.0)	23.4 (7.9)	
	Median (min, max)	19.7 (14.4, 36.3)	18.1 (14.4, 36.3)	19.4 (15.4, 22.4)	19.9 (16.4, 34.4)	
	Median (Q1, Q3)	19.7 (16.4, 22.4)	18.1 (15.4, 28.1)	19.4 (16.9, 21.4)	19.9 (17.4, 32.4)	
Weight (kg)	*N*	14	4	4	6	0.656
	Mean (SD)	56.5 (17.1)	59.3 (22.1)	49.4 (4.7)	59.3 (19.9)	
	Median (min, max)	48.7 (39.5, 92.0)	50.2 (44.8, 92.0)	48.4 (45.0, 56.0)	51.5 (39.5, 85.5)	
	Median (Q1, Q3)	48.7 (45.0, 56.0)	50.2 (46.1, 72.5)	48.4 (46.7, 52.2)	51.5 (45.0, 83.0)	
Sex	Female	14	4	4	6	-
Survival (days)	*N*	14	4	4	6	0.0754
	Mean (SD)	103.1 (51.1)	63.8 (66.2)	143.5 (38.9)	102.5 (26.5)	
	Median (min, max)	116.5 (6.0, 192.0)	61.5 (6.0, 126.0)	138.0 (106.0, 192.0)	105.0 (67.0, 133.0)	
	Median (Q1, Q3)	116.5 (80.0, 126.0)	61.5 (6.5, 121.0)	138.0 (112.5, 174.5)	105.0 (80.0, 125.0)	
Maximum ISP^b^ (mmHg)	*N*	14	4	4	6	< 0.0001
	Mean (SD)	51.9 (45.6)	114.5 (35.4)	39.3 (6.9)	18.7 (5.6)	
	Median (min, max)	34.0 (8.0, 155.0)	115.0 (73.0, 155.0)	38.5 (32.0, 48.0)	20.5 (8.0, 23.0)	
	Median (Q1, Q3)	34.0 (22.0, 73.0)	115.0 (87.0, 142.0)	38.5 (34.0, 44.5)	20.5 (18.0, 22.0)	
MRI^c^ (area, %)	*N*	13	4	4	5	< 0.0001
	Mean (SD)	35.4 (12.6)	51.3 (5.8)	33.3 (3.6)	24.5 (6.2)	
	Median (min, max)	32.9 (14.1, 57.5)	51.4 (44.8, 57.5)	33.1 (29.1, 37.9)	25.2 (14.1, 29.8)	
	Median (Q1, Q3)	32.9 (28.4, 44.8)	51.4 (46.5, 56.1)	33.1 (31.0, 35.6)	25.2 (25.0, 28.4)	
Time to recovery (days)		10	0	4	6	< 0.0001
	Mean (SD)	3.2 (4.2)	Nil	8.0 (0.8)	0.0 (0.0)	
	Median (min, max)	0.0 (0.0, 9.0)	Nil	8.0 (7.0, 9.0)	0.0 (0.0, 0.0)	
	Median (Q1, Q3)	0.0 (0.0, 8.0)	Nil	8.0 (7.5, 8.5)	0.0 (0.0, 0.0)	

a SD, standard deviation;

b ISP, intraspinal pressure;

c MRI, magnetic resonance imaging.

The mean observation period in the complete SCI group was 63.8 ± 66.2 (range, 6–126) days and two premature deaths were reported. Both the animals were completely paralyzed postoperatively and died within 7 days due to bladder rupture. High intraoperative spinal compression pressures of 73 and 101 mmHg were recorded in these two pigs. The mean observation period in the incomplete SCI group was 143.5 ± 38.9 (range, 106–192) days with a mean recovery period of 8 (range, 7–9) days. In the asymptomatic group, the mean observation period was 102.5 ± 26.5 (range, 67–133) days. Except for those two premature death animals, the rest of the pigs have undergone observation for at least 2 months.

### 3.2. Intraspinal pressure

**Figure 6:**
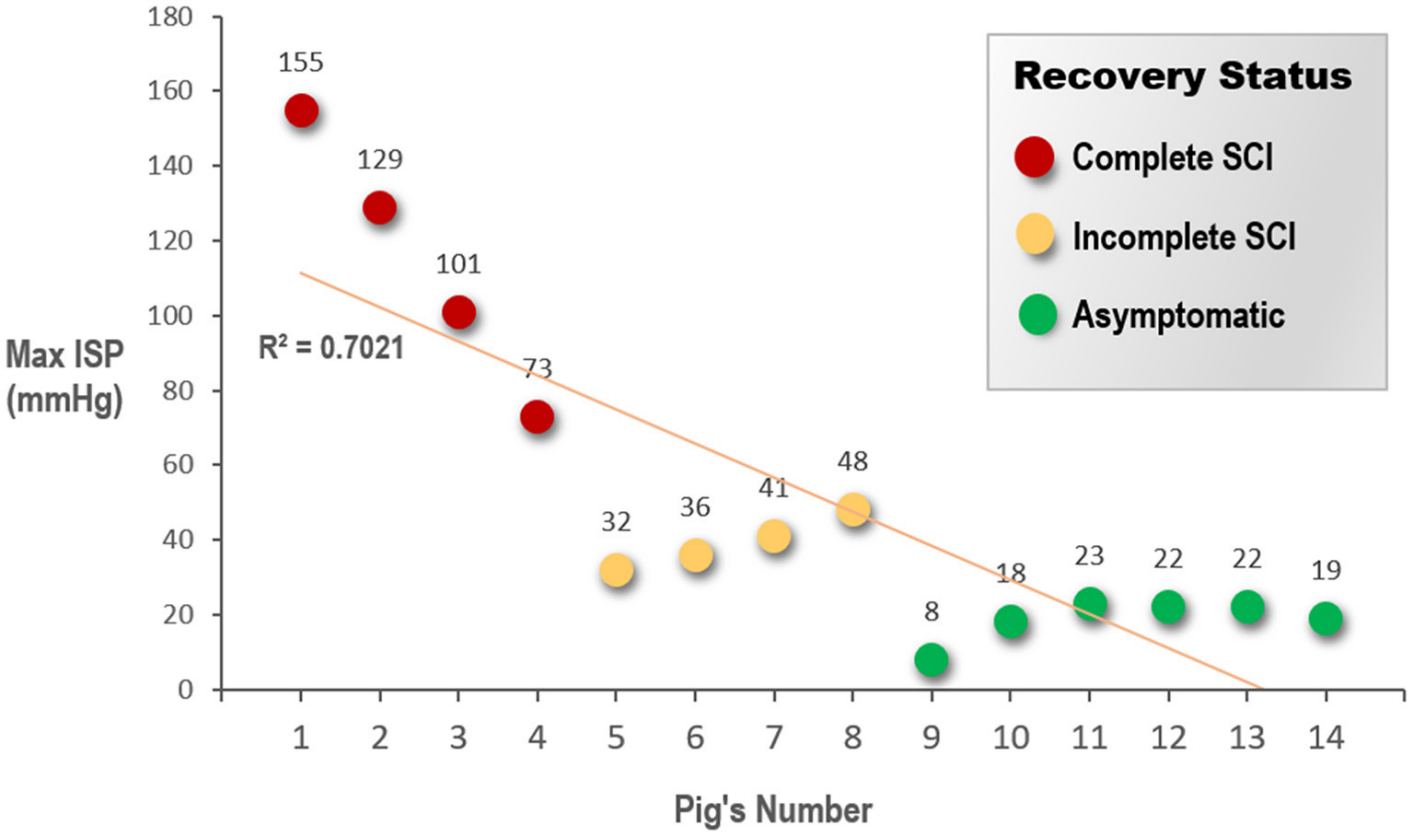
Pigs are grouped according to the recovery status of the animals' hind limbs after surgery. The graph shows a scatter plot to indicate the difference in the maximum ISP that the spinal cord suffered in each animal. ISP, intraspinal pressure; SCI, spinal cord injury.

The compression on the spinal cord was recorded using a pressure sensor in the epidural space. In all three groups, a normal baseline ISP was measured as 2–3 mmHg inside the spinal canal before compression. The mean maximal pressure was 114.5 ± 35.4 (range, 73–155) mmHg in the complete SCI group, which included two pigs that showed premature death, 39.3 ± 6.9 (range, 32–48) mmHg in the incomplete SCI group, and 18.7 ± 5.6 (range, 8–23) mmHg in the asymptomatic group (Figure 6); the between-group differences were statistically significant (*P* < 0.0001). The postoperative hindlimb performance of pigs represents different severity of spinal cord injury (from asymptomatic to complete paralysis). In the group with worse prognosis, the pressure applied during the experiment was higher. Especially, the greater the compressive pressure on the spinal cord, the more severe was the injury, and consequently, the poorer the postoperative hindlimb outcome of the pig.

### 3.3. Electrophysiologic data

SSEPs, MEPs, and SP-EPs were recorded intraoperatively. The SSEPs, MEPs, and SP-EPs were flattened in the complete SCI group when maximum pressure was achieved and did not partially return when the balloon was released. In the incomplete SCI group, SSEPs remained stable throughout the procedure. However, the MEP and SP-EP values dropped to a flat state when the pressure reached a maximum. After balloon deflation, the SP-EP amplitude returned to 20% of the baseline value in one pig and 50% in another, although the latency remained prolonged.

**Figure 7:**
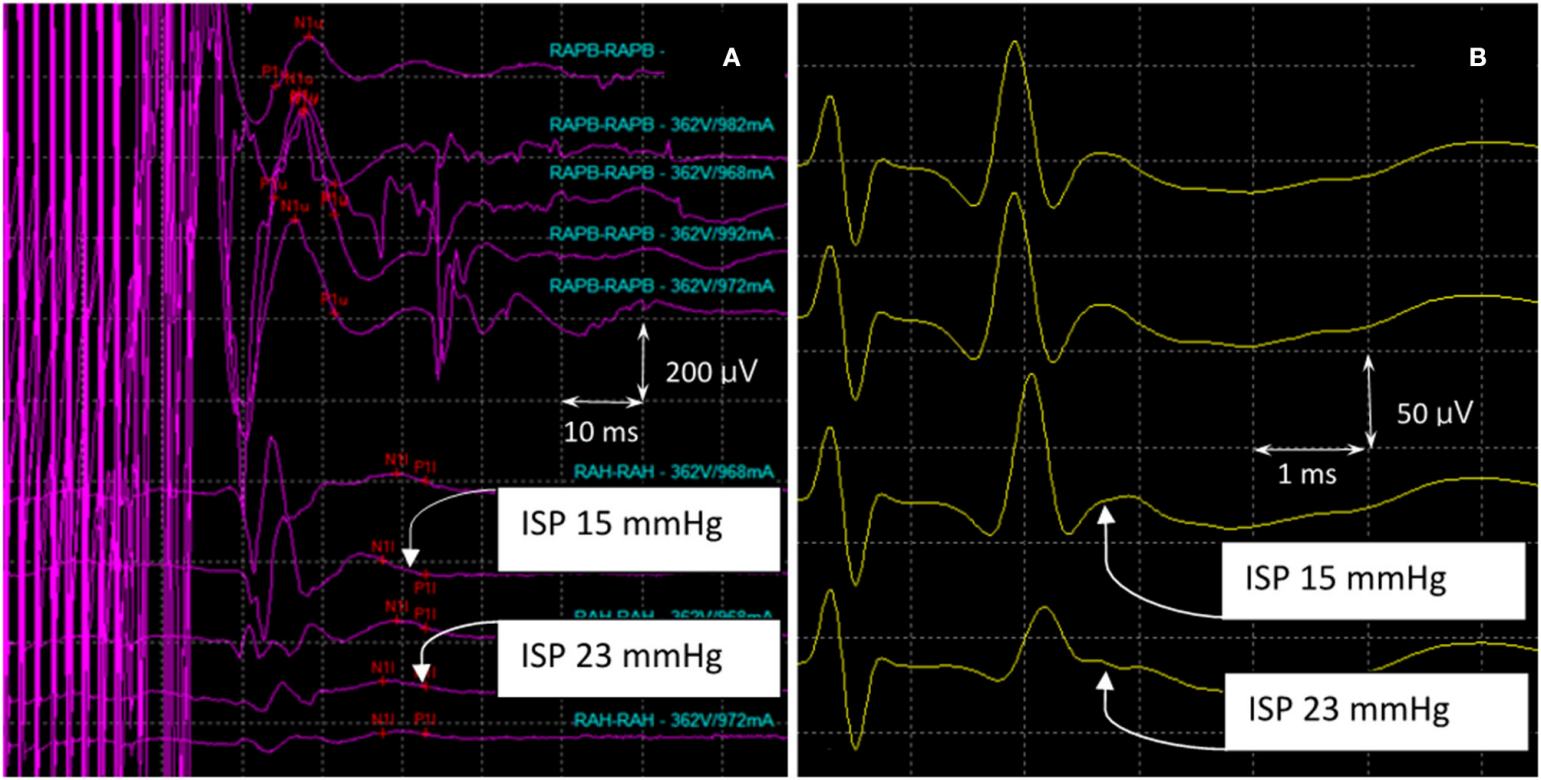
MEPs and SP-EPs were recorded in pig 11. **(A)** The waveforms (RAPB) in the upper part of the figure represent MEPs from the forelimb of the pig, which maintains a complete structure during the experiment. The waveforms in the lower part represented hindlimb MEPs (RAH). As the compression increases, the waveform declines gradually, resulting in a complete loss when the maximum compression pressure is reached. **(B)** As the compression increases, there is an initial prolongation of SP-EP latency which is followed by a decrease in amplitude. At this point, the balloon was released, and the amplitude decreased by 50% from the maximum pressure recorded. MEPs, motor evoked potentials; SP-Eps, spine-to-spine evoked spinal cord potentials.

**Figure 8:**
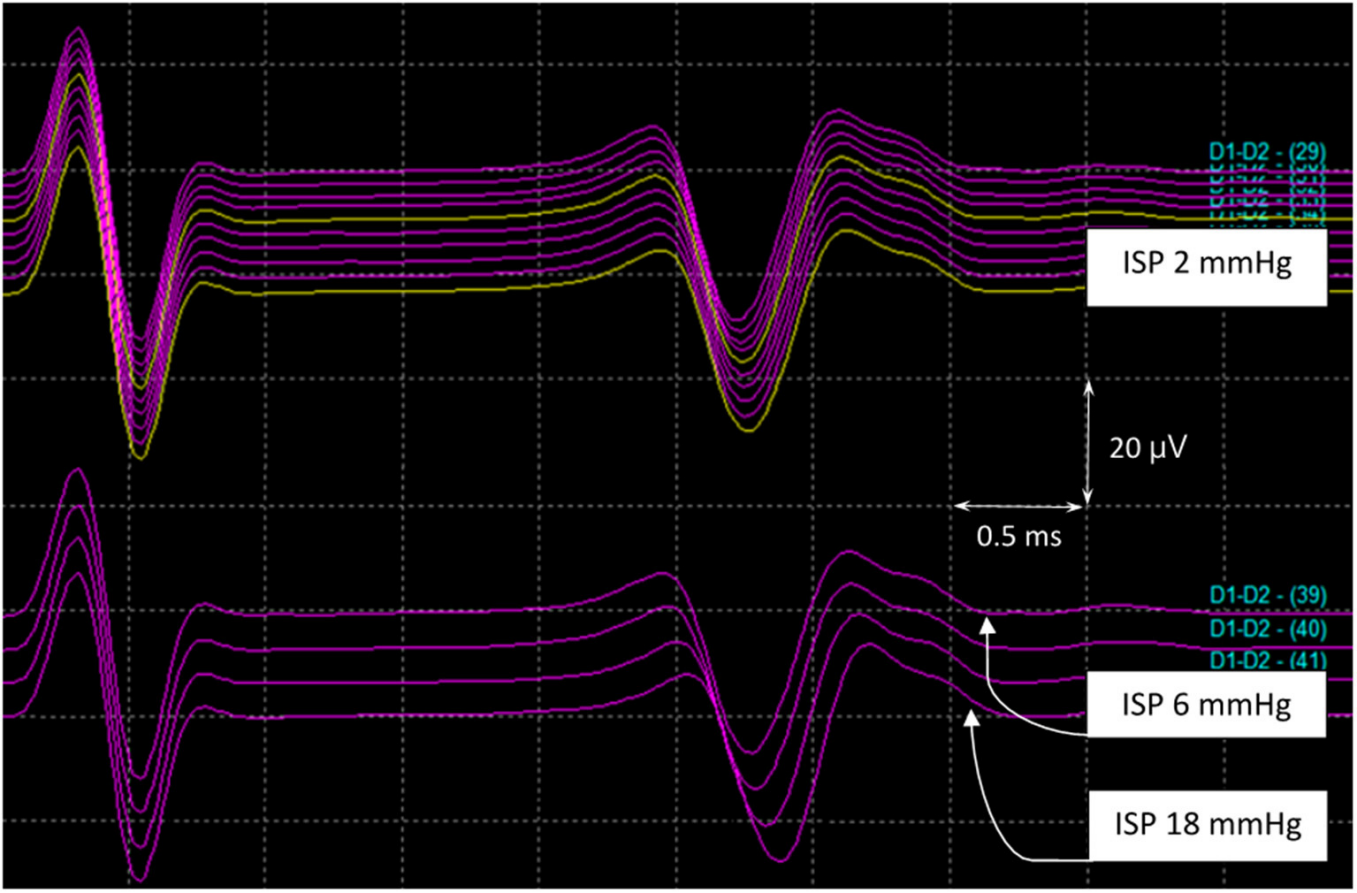
SP-EPs in pig 14. MEPs could not be tested under inhaled general anesthesia. The SP-EP latency started to become prolonged when the ISP reached 6 mmHg. At this point, inflation ceased, and the balloon was released at an ISP of 18 mmHg before the amplitude dropped. ISP, intraspinal pressure; MEPs, motor evoked potentials; SP-Eps, spine-to-spine evoked spinal cord potentials.

In the asymptomatic group, the SSEP values remained intact, except in pig 9, wherein the amplitude decreased by 40% of the baseline value. MEP amplitudes were maintained with complete loss of the SP-EP waveform. In pig 10, SP-EPs completely diminished when the MEPs dropped to 60%. After deflation of the balloon, there was no recovery of the waveform during wound closure. In pig 11, MEPs were lost completely, with SP-EPs preserved at a ratio of 50% (Figure 7). Inhaled anesthesia was administered to the remaining three pigs (pigs 12, 13, and 14). Although MEPs could not be tested under inhaled general anesthesia, the SP-EP waveform remained intact before compression. In pig 12, the SP-EP waveform decreased by 50% and we deflated the balloon. In pig 13, the SP-EP decreased by 70% but recovered partially after balloon deflation. In pig 14, when SP-EP latency started to increase, inflation was stopped, and the balloon was released immediately (Figure 8). None of the pigs had hindlimb weakness after compression, even without MEP monitoring.

In all SCI groups, prolongation of SP-EP latency appeared earlier than the decline in SP-EP amplitude. In most cases, latency prolongation occurred earlier than changes in MEP and SSEP waveforms.

### 3.4. MRI of the spinal cord

MRI examinations were performed on postoperative day 3 in each group. However, for one pig in the asymptomatic group, MRI was missed on the scheduled day as the MRI machine was under maintenance. We measured the high-intensity area ratio to the spinal cord's cross-sectional area. The average ratio was 51.3 ± 5.8% (range, 44.8–57.5) in the complete SCI group, 33.3 ± 3.6% (range 29.1–37.9) in the incomplete SCI group, and 24.5 ± 6.2% (range 14.1–29.8) in the asymptomatic group. A significant between-group differences was observed (*P* < 0.0001).

## 4. Discussion

We have created a SCI model consisting of 14 human-like porcine thoracic spinal cords using a balloon catheter at different levels of volume-induced gradient compression. A technique for measuring the exact pressure exerted on the spinal cord during compression was developed by attaching an ISP monitor to the balloon area, which allowed us to induce different degrees of spinal cord damage. We found a strong negative correlation between the intensity of pressure applied to the cord and functional outcomes. Especially, the ISP in the asymptomatic group was significantly lower than that in the transient paraplegic group and even in the complete SCI group (*P* < 0.0001). During surgery, neural monitoring techniques using SP-EPs can effectively and safely monitor spinal cord damage, thereby preventing severe postoperative complications. Postoperatively, the sensitivity of the ratio of the high-intensity area to the cross-sectional area of the spinal cord on MRI is a good predictor of recovery.

### 4.1. Animal model

Rodents comprise 90% of laboratory animals, and large animals, such as sheep, non-human primates, or pigs, comprise only 5% (9). However, the spinal cord diameter is 8–9 mm in an average human weighing 70 kg but only 2–3 mm in a typical rat weighing 275–325 g. Therefore, a large animal model is believed to more closely represent the scale and physiology of the human spinal cord. The pig spinal cord at T10/11 is ~7 mm in diameter and is surrounded by a layer of cerebrospinal fluid, making it a more clinically relevant animal model for testing the biodistribution and effect of therapies applied extradurally or infused intrathecally (4, 11). The vascular supply to the thoracic spinal cord in the pig is similar to that in humans; therefore, porcine models of ischemic thoracic paraplegia secondary to thoracoabdominal aortic aneurysm surgery are widely used (12–14). Given that ischemia is likely involved in secondary injury after traumatic SCI, similarities in the vascular supply to the spinal cord between humans and pigs are desirable (1). Therefore, we explored the human-like size of the porcine thoracic spinal cord and intrathecal space to measure the intracanal pressures associated with SCI.

The spinal contusion injury model, also called the weight-drop impact technique, is widely used to simulate acute traumatic SCI. Pressure sensors are included on the impactor tips of commercial instruments, such as the New York University/MASCIS device or Infinite Horizon impactor, to present the impact force. However, compared with the spinal cord, the cross-sectional area of the heavy hammer is still too large. Therefore, the relationship between the degree of damage and the force or pressure applied to the spinal cord is difficult to keep constant or precise. Moreover, various etiologies encountered clinically in humans deliver a sustained and progressive force to the spinal cord for a specific time period, unlike the contusion models that deliver a single instantaneous punctate impact to the exposed spinal cord. Therefore, the compression model is more suitable for representing SCI sustained by humans.

Compression can be achieved using a balloon, clip, or forceps (15). However, lesions are often not linear oppressive forces; therefore, clips or forceps cannot represent a real-life situation. The balloon-induced closed injury method was pioneered by Tarlov et al. (16–18) who determined the time limits for recovery after acute and gradual compression in dogs using a balloon compression device. These studies led to the development of a volume-dependent SCI compression model that could be used in rats, monkeys, cats, and ferrets. Laminectomy or laminotomy is generally performed, followed by the insertion of a balloon into the epidural or subdural space (19). In our model, the balloon was placed epidurally because most pathological lesions, including tumors, trauma, or even iatrogenic injury, are extradural. Purdy et al. and Fukuda et al. used a similar puncture method to implant a balloon into a dog's epidural space, which was expanded to a specific volume. The position of the balloon and degree of SCI were examined using MRI (20, 21) and histological analysis (22). However, MRI examination was performed immediately after compression rather than when the damage and swelling of the nervous system became apparent. Furthermore, these animal models of SCI are not assessed with intraoperative electrophysiological monitoring or pressure measurements. Although the authors found no evidence of injury distal from the area where the balloon was inflated, injury arising from the catheterization process is a concern (20).

Using our model, a balloon was introduced into the extradural space *via* small laminotomy and was advanced upward by two vertebral levels, where it was fixed along the spinal cord's dorsal surface. The cord was compressed inside a closed system to imitate an actual pathogenic situation. When the balloon catheter was implanted into the epidural space, no changes in pressure or neuroelectrophysiologic signals were noted, indicating stability within the central nervous system. Therefore, this method is advantageous over the puncture method reported in the literature. Results from experimental paradigms have suggested that a sex bias in traumatic neurological outcome exists that favors female individuals and appears to involve the advantageous or disadvantageous effects of the gonadal sex hormones estrogen and progesterone or testosterone, respectively (23, 24). Taking this into consideration, we selected experimental animals of the same sex to exclude the factor of sex. Therefore, we believe that our model is reliable, reproducible, and easy to apply when studying most SCI situations.

### 4.2. Intraspinal pressure

In humans, normal CSF pressure has been reported to be ~5–15 mmHg (25). Pressure elevated transiently to 10.5 and 40 mmHg was associated with Valsalva maneuver and coughing, respectively (26, 27). From the treatment guidelines for severe head trauma, it has been clearly defined that the upper limit of the intracranial pressure (ICP) that the brain tissue can withstand is below 20–22 mmHg. Once this value is exceeded, an irreversible damage may be caused in the brain function. Before reaching this critical point, therapeutic intervention must be made with administration of drugs for reducing brain swelling, hyperventilation, lowering body temperature, or even surgical craniectomy decompression. All treatments are performed to prevent the cerebral pressure from breaking through the critical value, improve patient survival, and reduce sequelae (7, 8). Regarding the spinal cord, which belongs to the same nervous tissue as brain, clear tolerable threshold has not been identified to date. Quantifying local ISP at the injured segment could be useful clinically, providing a means of determining the likelihood of further neurological deterioration and in turn, whether urgent medical intervention or surgical decompression are required.

Moreover, pigs are quadrupeds, and their spines are horizontal; therefore, the spines are different from those of upright human beings. However, when human patients undergo spine surgery, no matter whether they are in the supine, prone, or even decubitus position, their spine is in a horizontal state, which is similar to that of the experimental animals. Therefore, we believe that the experiment can represent the state of human patients. After surgery, human patients lie flat in the acute stage, and the pressure in the spinal cavity can be obtained, similar to measuring the intracranial pressure in patients after brain surgery.

Most balloon compression models provide different degrees of damage by varying the balloon volume. However, even in the same species, the diameter and area of the spinal canal show a degree of interindividual variation. We believe that an exact pressure can represent the actual injury sustained by the spinal cord. Some weight drop-based or compression devices have a pressure sensor inside the impactor to measure the pressure delivered to the cord (1, 28, 29). However, they do not represent the actual pressure within the cord itself.

To the best of our knowledge, only a few studies have examined the relationship between spinal pressure and SCI under specific circumstances. Wolfla et al. (30) performed anterior corpectomy at C3 and placed a flexible ventral graded compression device incorporating a pressure transducer in cats weighing 2.6–5.0 kg. The implant was advanced in a stepwise manner into the epidural space of the spinal canal. Cervical ventral epidural pressure (CVEP) was measured at each degree of canal compromise and during neck movement in the flexed, extended, and neutral positions. Laminectomy was performed in some cats and was found to lower the CVEP under all conditions examined. The device displayed pressure changes in the spinal canal according to cervical spine posture and established that decompression surgery reduced compression on the spinal cord (30). This was a pioneering study of a compression model related to ISP. However, the device used was relatively bulky, and the pressure could not be measured following removal of the device. No functional outcomes could be evaluated in this model because the animals were sacrificed immediately after surgery.

Batchelor et al. developed a weight-drop-based rodent catheter model of SCI, in which there was an initial contusion injury followed by sustained compression with a varying-sized epoxy spacer. Pressure was measured using a water manometer attached to a fine catheter positioned centrally within each spacer. They found that the mean ISP was ~20 mmHg at 40% canal occlusion, >30 mmHg at 45% canal occlusion (which induced rapid neurological deterioration), and >40 mmHg at 60% canal occlusion. These pressures were substantially higher than the mean baseline epidural pressure of 4.5 ± 1.2 mmHg (31).

This study used a commercial ICP monitor mounted on a balloon. When the balloon is inflated, the pressure change occurs in real time and is quantifiable with baroreceptors attached directly onto the balloon. The pressure data directly represent the force on the SCI segment. The objective of our study was to establish an *in vivo* porcine model of SCI suitable for studying changes in pressure magnitude at the exact location where damage occurs during an SCI event.

We found a negative correlation between the maximal pressure applied to the spinal cord and the motor outcome; our results revealed that the higher the maximal ISP during operation, the more severe the motor outcome. The pressure sensor was placed *in situ* postoperatively, and the ISP and motion of the hindlimb were continuously recorded. On postoperative day 3, the pressure reached a plateau and slowly reverted to the initial value. The critical point between recoverable and unrecoverable SCI appears to lie between 20 and 30 mmHg. These preliminary data are similar to the critical points for human brain damage.

### 4.3. Evoked potentials and SP-EPs

The most widely used intraoperative neurophysiological monitoring techniques are upper and lower extremity SSEPs and transcranial MEPs, closely followed by pedicle screw simulation, and spontaneous electromyography (32). Several reports have indicated that SSEPs are preserved during a procedure in some patients who develop postoperative paraplegia (33–35). This is because the SSEP represents the function of the dorsal column rather than that of the anterior column, especially in the corticospinal tract. A combination of muscle MEPs and D-waves can better predict the functional outcome. It has been demonstrated that a preserved D-wave up to 50% of the original amplitude with a complete loss of muscle MEPs results in only transient paraplegia (36–38). Since muscle MEPs are extremely sensitive to inhaled anesthetics, muscle relaxants, and intraoperative convulsions, total intravenous anesthesia with propofol is preferred when monitoring muscle MEPs but renders surgery unstable and unsafe. The D-wave is purely a nerve action potential and does not involve synaptic activity; therefore, it is relatively insensitive to the effects of anesthesia. However, D-wave monitoring involves a risky insertion technique and cannot be used during surgery on the cord caudal to T10–11, where the corticospinal tract fibers become fewer, eventually disappearing in the lumbosacral region (39).

Costa et al. inserted two catheter electrodes into the epidural space both cranial and caudal to the surgical site, elicited a D-wave by TES, and obtained recordings at the spinal electrodes. The anti-D-wave evoked by epidural stimulation of the spinal cord with the same catheter electrodes was also recorded over the scalp at the midline. They found that in neurologically compromised patients, the anti-D-wave and D-wave exhibited similar behavior, both of which are present in neurologically intact or moderately compromised patients and absent in those with quadriplegia/paraplegia (40). Thus, we have confirmed that the D-wave electrode can be applied to the spinal cord for stimulation or reception, showing antegrade or retrograde electrophysiological signals in the spinal cord.

The SP-EP technique refers to stimulation of the spinal cord with an epidural catheter-type electrode whereby the elicited compound potentials are recorded over the spinal cord (41). Stimulation can be performed cranially and recorded caudally or vice versa. The recorded potentials are robust and most likely represent the combined activity of the dorsal columns, corticospinal tracts, and other tracts in the spinal cord. Ueta et al. (42) found that SP-EPs degraded earlier than MEPs or SSEPs, regardless of whether the cord compression came from the anterior, posterior, lateral, or circumferential direction. Unfortunately, Koyanagi et al. (43) could not find a clear correlation between the use of this method and the clinical outcomes in 20 patients who underwent surgery for intramedullary spinal cord tumors. However, in later studies, the SP-EP technique was used successfully to monitor spinal cord ischemia and function during aortic dissection surgery (44, 45). Moreover, this monitoring technique is unaffected by general anesthesia or muscle relaxants and can be used to monitor most segments of the spinal cord.

The findings of our preliminary study are as follows. First, SSEPs remained intact throughout the procedure in the incomplete SCI group, indicating a high false-negative rate of SSEPs. Second, when the SP-EP changed, latency prolongation was first initiated, followed by a decrease in SP-EP amplitude; if the compression was stopped immediately, the pigs did not lose strength. When the changes in SP-EPs were significant, but before the MEP disappeared, the pig experienced short-term paralysis. When both SP-EPs and MEP were lost, the pig was either transiently or permanently paralyzed. Finally, SP-EPs are resistant to the effects of anesthesia and allow neuromuscular blockade for paralysis. Therefore, given the strong correlation between SP-EPs and the motor status, coupled with the ability of these potentials to withstand general anesthesia, SP-EPs can be expected to have broad clinical applications. Even if patients are under general anesthesia, IONM is still reliable. When SP-EP latency is prolonged, the spinal cord may be compromised and requires intervention. When the SP-EP amplitude gradually declines, the spinal cord should be rescued. Even if SP-EPs are lost, the pig may experience transient weakness if the waveform shows partial recovery after the intervention.

### 4.4. MRI study

The quantitative properties of signals from myelons and spinal cord compression on MRI scans have been studied (10, 46, 47). However, most studies focused on the correlation between extreme outcomes, such as complete paralysis, and the presence of hyperintensity on MRI, especially along the length of hyperintense lesions. In a homogenous dog population with thoracolumbar disc herniation, Boekhoff et al. (48) evaluated the presence and degree of spinal cord compression by comparing the cross-sectional diameter of the spinal cord at the site of disc herniation with the cross-sectional diameter of the spinal cord at one vertebra caudal to the herniation on transverse T2W images. If an intramedullary hyperintensity was present on T2W images, the length was measured on sagittal T2W images and divided by the length of the L2 vertebra to create the T2W length ratio described by Ito et al. (48, 49). They reported a significant correlation between the quantitative characteristics of T2W images and outcomes. We believe that the lengths of hyperintense lesions and hemorrhage are not always easily defined. As the appearance of syringomyelia on MRI also has high signal intensity, it easily mimics spinal cord edema or hemorrhage. By observing the relationship between the MRI images and clinical neurological deficits, we believe that it is more critical to evaluate the degree of spinal cord compression by calculating the ratio of the hyperintensity area to the total cord cross-sectional area at the maximally damaged segment instead of the normal segment.

Recent human studies have analyzed the predictors of MRI findings, which were correlated with neurologic outcomes in patients with acute cervical SCI. There was a statistically significant correlation between the extradural finding of maximal spinal cord compression, intradural MRI findings of the length of cord edema, and length of intramedullary hemorrhage with neurological recovery in patients with sustained trauma (50–52).

According to a literature review, the timing of *in-vivo* MRI examinations varies from intraoperatively to 4 h, 3, 10 days, 3–4 weeks, 2 months, or even 1 year after injury (20–22, 53–56). To the best of our knowledge, most patients with head injury experience brain swelling in the first week after trauma, especially on day 3. From our ISP recording, a similar pressure curve was found, in which the highest ISP was recorded on day 3 after injury and, then, declined gradually on the days following. Therefore, we performed spinal MRI on day 3 after SCI induction.

In our study, animals with complete SCI had the largest spinal cord edema area, which was roughly estimated to be more than 50%. The average area of incomplete SCI was approximately 33%, and the area of cord edema in the asymptomatic group was < 25%. Our findings confirmed that this ratio was strongly correlated with the prognosis. To the best of our knowledge, no MRI studies have been conducted to subdivide the degree of cord compression from minor to severe. Pigs with evidence of spinal cord damage on imaging studies, but without postoperative neurological deficits, were also included in our study to demonstrate the limits of injury that the spinal cord could withstand.

### 4.5. Limitations and future possibilities

Despite meaningful results and potential translational value from our paper, admittedly, the study has limitations. First, our sample size was relatively small. The initial and continued cost of maintenance of pigs is much higher. Long-term care difficulties, such as catheter management and development of pressure sores, further limits our number. Although we believed our study provides important information regarding spinal cord injury, increasing the sample size may provide more reliable and reproducible data to ensure credibility. Second, our model did not represent all SCI mechanisms. Characteristic mechanisms of clinical SCI include: (i) contusion impact plus persistent compression of the spinal cord; (ii) impact alone with transient compression; (iii) distraction; and (iv) laceration/transection (57). We deliberately slowed the rate of balloon inflation to observe the ISP slope and electrophysiological changes. However, acute impact, not to mention distraction or laceration, may not have been achieve in this model. Accelerating the compression speed to mimic a sudden traumatic impact to the cord or slower compression to simulate slow-growing spinal tumors should be included in future studies. Third, SCI in humans is most frequently determined by structures located ventral to the cord, such as the intervertebral disc or the vertebral bodies, while in our experimental settings, the compression force was applied dorsally. In addition, even though we used fluoroscope to confirm the balloon location, the epicenter of the SCI within the transverse area of the spinal cord was not 100% guaranteed. If we can effectively control the compression position, we can extend our research setting to simulate more injury modes such as anterior column syndrome or brown-sequard syndrome. Fourth, we observed our pigs for 3.4 months on average, with ranges from 6 days to 6.4 months. In previous studies and clinical observation, spontaneous recovery of SCI in humans is not considered to reach a plateau until 6–12 months after injury. The recovery of rats, however, typically plateaus at 6–8 weeks after injury (58). There was no sufficient data on the recovery plateau in pigs from previous studies, and longer observation periods may be needed. Fifth, we could only descriptively analyze the neurophysiological results without effective statistical verification in present study. And as we mentioned before, the small sample size made gathering enough data difficult. Additionally, during gradient compression, evoked potential changes often occur instantaneously, and we cannot precisely stop. However, some interesting trends can be seen, especially for SP-EPs that can ignore general anesthesia and muscle relaxant agents and is sensitive enough. More samples and whether the amount of compression should be controlled more finely in future experiments should be considered. Sixth, we did not use the injured spinal cord's histopathological findings to evaluate the degree of damage. We agreed with the literature suggestion that MRI provided high-resolution images of tissue SCI, which correlated closely with the injury volume seen by histopathology, which included intramedullary hemorrhage, inflammation, necrosis, and apoptosis (59). Outcome prediction or treatment efficacy is not defined by autopsy in human patients with SCI. In our opinion, the use of electrophysiology, ISP, and imaging to correlate or predict recovery after injury is much closer to the reality of human SCI. However, we also fully agree it is absolutely necessary to obtain histopathological findings in order to evaluate the mechanism and effect of the treatment.

Even with limitations, we believe that our model has achieved effective results and has the potential to expand the development and application in the future. Yang et al. inflated “balloons” that were left in the spinal canal for up to 4 weeks post-surgery to mimic the chronic compression mechanism, as a tumor or infection does (60). By combining our ISP monitoring method, spinal canal pressure can be implemented long term and followed simultaneously. A study for the optimal timing of an intervention can be designed, just like the importance of ICP in a traumatic brain injury. Moreover, the importance of the focal partial pressure of O_2_ (PaPO_2_) (61), blood flow (62), metabolites in the CSF, and spinal perfusion pressure have gained attention. A pig's spinal cord is large enough to allow the probes to be inserted and evaluated. This information will be useful for translating the information into the clinic with respect to patient care, monitoring, and management practices (63). With collecting more data, we should be able to integrate these clinical parameters so a scoring system can be achieved for predicting the prognosis.

## 5. Conclusion

We used pigs that were similar to humans in terms of weight and spinal structure, as a model of SCI. In addition to conventional IONM of SSEPs and MEPs, we introduced SP-EPs for more specific monitoring of subtle electrophysiologic changes at the lesion site. SP-EPs are resistant to the effects of anesthesia and neuromuscular blockade. Changes in the shape and amplitude of SP-EPs were significantly related to paraplegic severity in pigs after SCI. The latency prolongation of SP-EPs seems to be the most sensitive warning sign that emerges much earlier than that of MEP or SSEP. Introducing a novel ISP monitoring technique meant that the spinal pressure could be quantified in real-time. The higher the maximal ISP during operation, the more severe the motor outcome. The ratio of the hyperintense area to the total spinal cord cross-sectional area at the segment with sustained maximum damage on spinal MRI after SCI also played a role in predicting the outcome. By integrating SP-EPs, spinal cord pressure, and findings on MRI, we built a simple, real-time monitoring, and predictable system that can detect impending SCI or early iatrogenic trauma to improve the treatment outcomes. However, experiments with larger sample sizes are required to further validate our findings.

## Data availability statement

The raw data supporting the conclusions of this article will be made available by the authors, without undue reservation.

## Ethics statement

The animal study was reviewed and approved by Institutional Animal Care and Use Committee of Pigmodel Animal Technology Co., Ltd.

## Author contributions

C-KH and M-HC initiated the study and designed the experiments. C-KH performed the surgical procedures, analyzed the data, and wrote the paper. Y-HW established and performed the anesthesia procedures, postoperative care, and recordings. C-YW obtained the intraoperative electrophysiology measurements. J-SS and C-YW supervised the study and critically revised the manuscript. All authors read and approved the final manuscript.
